# Efficacy of Sucralfate-Combined Quadruple Therapy on Gastric Mucosal Injury Induced by *Helicobacter pylori* and Its Effect on Gastrointestinal Flora

**DOI:** 10.1155/2020/4936318

**Published:** 2020-08-31

**Authors:** Guigen Teng, Yun Liu, Ting Wu, Weihong Wang, Huahong Wang, Fulian Hu

**Affiliations:** ^1^Departments of Gastroenterology, Peking University First Hospital, Beijing, China; ^2^Department of Gastroenterology, Peking University People's Hospital, Beijing, China

## Abstract

**Background:**

This study explored the therapeutic efficacy of standard triple therapy combined with sucralfate suspension gel as well as the mechanisms of action in mouse models of *H*. *pylori* infection.

**Materials and Methods:**

C57BL/6J mice were randomly divided into 5 groups: NC (natural control), HP (*H*. *pylori* infection), RAC (rabeprazole, amoxicillin, and clarithromycin), RACS (RAC and sucralfate suspension gel), and RACB (RAC and bismuth potassium citrate). HE staining and electron microscopy were performed to estimate histological and ultrastructural damages. The IL-8, IL-10, and TNF-*α* of gastric antrum tissues were measured by immunohistochemistry and qRT-PCR. ZO-1 and Occludin were also detected with immunohistochemistry. The genomes of gastric and fecal microbiota were sequenced.

**Results:**

The eradication rate of *H*. *pylori* in the RACS group was higher than the RAC group. RACS therapy had protective effects on *H*. *pylori*-induced histological and ultrastructural damages, which were superior to the RAC group. RACS therapy reduced the protein and mRNA levels of IL-8 compared with the RAC group. The expression of Occludin in the RACS group was significantly higher than that of the RAC group. The composition of gastric and fecal microbiota for RACS was similar to the RACB group according to PCA.

**Conclusions:**

The RACS regimen eradicated *H*. *pylori* infection effectively and showed RACS had protective effects against *H*. *pylori*-induced histological and ultrastructural damage. The mechanisms of RACS effects included decreasing IL-8, enhancing Occludin, and transforming gastric microbiota. Moreover, RACS and RACB have a similar effect on gastrointestinal flora.

## 1. Introduction

*Helicobacter pylori* (*H*. *pylori*), a Gram-negative, microaerophilic bacterium, is closely associated with chronic gastritis, peptic ulcers, and gastric adenocarcinoma [1]. This bacterium is able to colonize and survive in the gastric environment by several mechanisms, including the adherence to the epithelium and breakdown of urea with production of ammonium which neutralizes the gastric acidity [1]. Improving the eradication rate of *H*. *pylori* is particularly important due to the high rates of *H*. *pylori* infection and high morbidity of gastric cancer in China [2, 3]. The eradication rates of standard triple therapy have been declining due to increased antibiotic resistance [4, 5]. In China, bismuth-containing quadruple therapy is currently the recommended first-line treatment [6], but its administration is limited due to adverse effects of bismuth.

Recently, the efficacy of gastric mucosal protective agents in *H*. *pylori* eradication has been widely estimated. Several mucosal protective agents combined with PPI+antibiotic therapy have been validated to increase eradication rates and reduce side effects [7–12]. Sucralfate suspension gel (SC) is a sucrose sulfate compound [13]. Its clinical efficacy for ulcer and chronic gastritis has been observed, but the role of sucralfate suspension gel for eradicating *H*. *pylori* has not been determined. The mechanisms of nonantibiotic drugs to eliminate *H*. *pylori* can be summarized as decreasing inflammatory factors, enhancing the mucosal barrier, transforming gastric microbiota, and so on [14].

In this study, we tried to explore the effect of standard triple therapy+sucralfate suspension gel (rabeprazole, amoxicillin, and clarithromycin and sucralfate suspension gel (RACS)) on *H*. *pylori*-induced histological and ultrastructural damages, inflammatory factors, tight junction protein, and gastric and fecal microbiota in *H*. *pylori* infection mouse models.

## 2. Materials and Methods

### 2.1. Experimental Animals, Medicine, and Strains

Male C57BL/6J mice at an age of 6-8 weeks and weight of 18~22 g were purchased from SPF Biotechnology Company (Beijing, China). *H*. *pylori* Sydney strain 1 (SS1) was cultured for *H*. *pylori* infection mouse models. SC was provided by Kunming Jida Pharmaceutical Co. Ltd. The experiment was approved by the Animal Ethical Committee of the First Hospital of Peking University (No. J201819).

### 2.2. Animal Model of *H*. *pylori* Infection and Treatments

After adaption to their environment for 1 week, mice were randomly divided into 5 groups: natural control group (NC, *n* = 6), *H*. *pylori* infection group (HP, *n* = 12), standard triple therapy group (rabeprazole, amoxicillin, and clarithromycin (RAC), *n* = 12), standard triple therapy and sucralfate group (RAC and SC (RACS), *n* = 12), and bismuth-containing quadruple therapy group (RAC and bismuth potassium citrate (RACB), *n* = 12). Except for the NC group, mice were given doses of 1 × 10^9^ CFU SS1 in 0.2 ml brucella broth by oral gavage (every other day, 5 times total). After postinfection, one mouse in each group was killed randomly and *H*. *pylori* colonization was confirmed by immunohistochemical staining. One week after *H*. *pylori* infection, the RAC group was fed with 4 mg/kg omeprazole, 206 mg/kg amoxicillin, and 103 mg/kg clarithromycin. Based on standard triple therapy for the RAC group, 206 mg/kg SC was added in the RACS group and 123 mg/kg bismuth was used simultaneously in the RACB group (twice daily for 14 days). Animals in the NC and HP groups were given the same volume of normal saline. All groups of mice were sacrificed on six days after the last administration of eradication therapies. *H*. *pylori* colonization was tested by immunohistochemical staining and PCR (the sequences are shown in Supporting Information Table 1). Gastric tissues were cut lengthwise in order to observe both the antrum and the body simultaneously and to confirm *H*. *pylori* on 30 slides unless positive result was observed.

### 2.3. HE Staining and Electron Microscopy

Gastric tissues were fixed by 10% neutral formalin, embedded in paraffin, and cut into 4 *μ*m thick sections. The sections were stained with standard hematoxylin and eosin (HE) staining by Liu Y and independently scored by two blinded investigators (Teng GG and Wu T). The score for epithelial damage (EDS) was based on the following criteria: 1 (normal mucosa), 2 (mucosal surface cell damage), 3 (glandular cell damage), and 4 (erosion, bleeding, or ulcers). The ultrastructure of the gastric antrum was observed with transmission electron microscopy.

### 2.4. Immunohistochemical Staining (IHC)

After deparaffinization and rehydration, the endogenous peroxidase activity in the sections was inhibited with 3% hydrogen peroxide. Then, the slides were transferred in antigen retrieval and incubated with a primary antibody at 4°C overnight. After incubation with a secondary antibody at room temperature for 1 hour, 3,3′-diaminobenzidine was used. Following counterstaining with hematoxylin, the sections were observed under an optical microscope. The primary antibodies including rabbit anti-*H*. *pylori* (1 : 250), rabbit anti-mouse interleukin-8 (IL-8, 1 : 80), rabbit anti-mouse tumor necrosis factor-*α* (TNF-*α*, 1 : 150), rat anti-mouse IL-10 (1 : 100), rabbit anti-mouse zonula occludens-1 (ZO-1, 1 : 150), and rabbit anti-mouse Occludin (1 : 100). The antibodies were purchased from Abcam (Cambridge, UK) or Absin (Shanghai, China). The IHC procedure was performed by Liu Y, and immunostaining scores were completed independently by two blinded investigators (Teng GG and Wu T). Five fields of vision (at ×400) per section (2 to 3 sections per specimen) were scored. The staining intensity was scored as follows: 1 (negative, brown), 2 (weak brown), 3 (moderate brown), and 4 (strong brown). The extent of staining was based on the percentage of positive cells: 0 (0-5%), 1 (6-25%), 2 (26-50%), 3 (51-75%), and 4 (76-100%). The final score was defined as the sum of the intensity and extent scores.

### 2.5. RNA Extraction and Real-Time Quantitative PCR

Total RNA from gastric tissues was isolated using TRIzol reagent. Then, a Reverse Transcriptase Kit (TaKaRa Biotechnology Group, Dalian, China) was used to generate cDNA. qRT-PCR was conducted on the Applied Biosystems 7500 Real-Time PCR System with SYBR Green Master Mix (Thermo Fisher Scientific, Grand Island, NY, USA). The primer sequences are displayed in Supporting Information Table 1.

### 2.6. 16S rRNA Gene Sequence

Total DNA was extracted from gastric tissues (antrum and body) and fecal samples using the QIAamp PowerFecal DNA Kit (Qiagen, Hilden, Germany). The genomic DNA was examined with a NanoDrop 2000 spectrophotometer and 1% agarose gel electrophoresis to confirm concentration, integrity, and size. The V3-V4 region of bacterial 16S rRNA genes was amplified using universal primers (341F and 806R) linked with indexes and adaptors. Then, these amplicons were sequenced on a HiSeq platform (Illumina, Inc., CA, USA) for paired end reads of 250 bp. DNA extraction and sequencing were conducted at Realbio Genomics Institute (Shanghai, China).

### 2.7. Statistical Analysis

The data were analyzed with SPSS 21.0 and R software. Continuous variables were displayed as means and standard deviations. To investigate whether differences among different groups are statistically significant, data were analyzed by one-way analysis of variance (ANOVA) followed by the Tukey test or Kruskal-Wallis test and followed by the Nemenyi test for multiple groups. The eradication rate was calculated using Fisher's exact test. The sequencing analyses for gastric microbiota or fecal microbiota were conducted with R software. *P* values < 0.05 were defined as statistically significant.

## 3. Results

### 3.1. RACS Therapy May Be Superior to RAC Therapy for *H*. *pylori* Infection in Mice

The colonization rate of *H*. *pylori* in the HP group was 91.67% (11/12), while it was 0.00% (0/6) in the NC group. The eradication rates were 66.67% (8/12) in the RAC group, 83.33% (10/12) in the RACS group, and 91.67% (11/12) in the RACB group. These results suggest that eradication efficacy of RACS may be effective, which still requires further clinical trials.

### 3.2. Protective Effects of RACS on *H*. *pylori*-Induced Histological and Ultrastructural Damages

**Figure 1:**
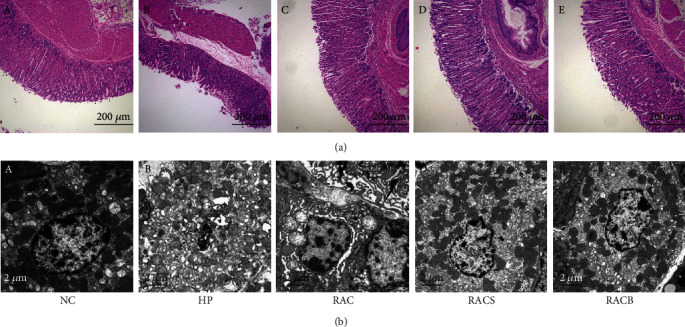
RACS attenuated *H*. *pylori*-induced histological and ultrastructural damages. (a) Typical microscopic images by HE staining of gastric mucosa: (A) natural control group (NC), (B) HP model group (HP), (C) standard triple therapy group (RAC), (D) standard triple therapy+sucralfate group (RACS), and (E) bismuth-containing quadruple therapy group (RACB). Scale bar: 200 *μ*m. (b) Transmission electron micrograph images: (A) NC, (B) HP, (C) RAC, (D) RACS, and (E) RACB. Scale bar: 2 *μ*m.

As shown in Figure 1(a), there were intact structures of gastric mucosa without inflammatory cell infiltration in the NC group. The gastric mucosal epithelium was unclear, and erosion or ulcers were observed in the HP group. The histological damage for three treatment groups was attenuated with mucosal surface cell damage along with mild inflammatory cell infiltration. The EDS is shown in Supporting Information Table 2. The EDS in the RACS group was significantly decreased compared with that in the RAC group (*P* = 0.019) and was lower than that in the RACB group without significance (*P* = 0.382).

The ultrastructure of the gastric antrum is shown in Figure 1(b). The normal cell structures and abundant secretory granules were seen in the NC group. The mitochondria and endoplasmic reticulum were swollen with sparse microvilli and decreased secretory granules in the HP group. The ultrastructural damage of three treatment groups was reduced, which was similar to the NC group, but swollen mitochondria were still seen in the RAC group. These data showed that RACS therapy has protective effects against *H*. *pylori*-induced histological and ultrastructural damages.

### 3.3. RACS Inhibited the Overexpression of IL-8 Induced by *H*. *pylori*

The mRNA levels of IL-8 (also known as chemokine ligand 15 (Cxcl15)), IL-10, and TNF-*α* expression were upregulated in the HP group compared with the NC group (*P* = 0.017; *P* = 0.247; and *P* = 0.038). Additionally, the IL-8, IL-10, and TNF-*α* mRNA levels significantly decreased after different eradication therapies. The IL-8 mRNA level of the RACS group was significantly lower than that of the RAC group (*P* = 0.041) but similar to that of the RACB group (*P* = 0.988). No significant differences were found in IL-10 or TNF-*α* mRNA levels between the RACS and RAC therapies (*P* = 0.136; *P* = 0.975). The protein levels of IL-8, IL-10, and TNF-*α* followed the same trend (Table 1, Supporting Information Figure 1). These data indicate that RACS therapy induces an anti-inflammatory response, especially by reducing IL-8.

### 3.4. RACS Enhanced Expression of the Tight Junction Protein Occludin

As shown in Table 2 and Supporting Information Figure 2, ZO-1 and Occludin IHC scores were downregulated in the HP group compared with the NC group (*P* = 0.009; *P* < 0.001). The expression of ZO-1 and Occludin in the three treatment groups was higher than that in the HP group. No significant difference was noted in ZO-1 protein levels between the RACS and RAC therapies (*P* = 0.961). The Occludin expression for the RACS group and RACB group was elevated significantly compared with that for the RAC group (*P* < 0.001). The data suggest that RACS significantly enhanced expression of Occludin.

### 3.5. Alteration of Gastric Microbiota Composition during Eradication Therapy in *H*. *pylori*-Infected Mice

#### 3.5.1. Composition of the Gastric Microbiota

**Figure 2:**
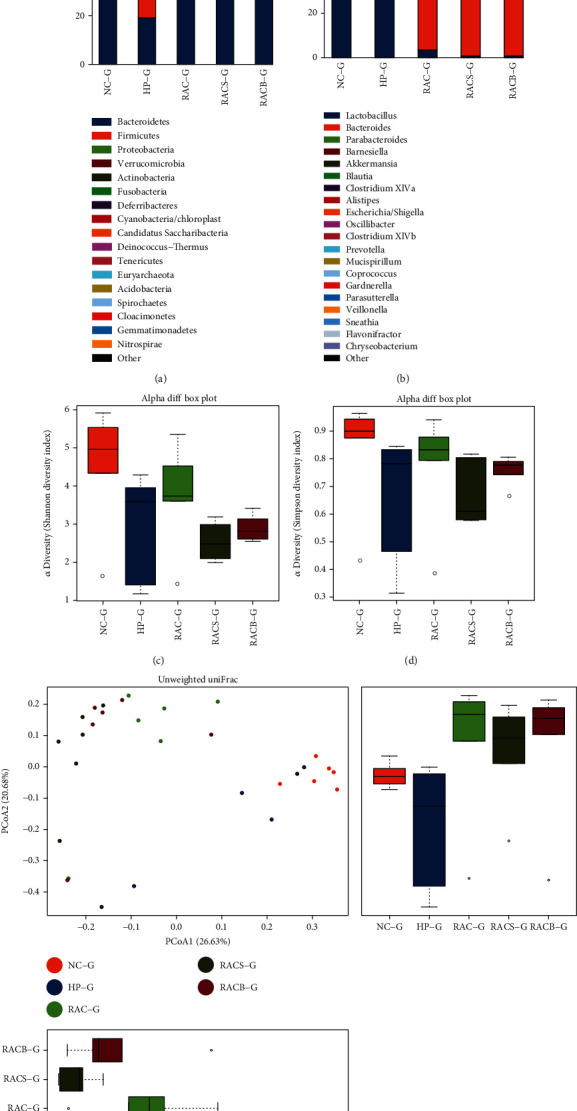
The composition, alpha diversity, and beta diversity of the gastric microbiome in mice. (a) Relative abundance distribution of major phyla of gastric microbiota composition in each group. (b) Relative abundance distribution of major genera of gastric microbiota composition in each group. (c) Shannon index of gastric microbiota based on the OTU counts. (d) Simpson index of gastric microbiota based on the OTU counts. (e) Unweighted PCoA of gastric microbiota. NC: natural control group; HP: *H*. *pylori* model group; RAC: standard triple therapy group; RACS: standard triple therapy+sucralfate group; RACB: bismuth-containing quadruple therapy group; G: gastric microbiota; OTU: operational taxonomic unit; PCoA: principal coordinate analysis.

A total of 871,470 clean reads with an average of 29,049 reads per sample were generated from 30 gastric tissues, and 819 OTUs at a 97% similarity level were generated afterwards. The most abundant phyla in the gastric tissues were *Bacteroidetes*, *Firmicutes*, *Proteobacteria*, and *Verrucomicrobia* with average relative abundances of 48.69%, 41.57%, 5.65%, and 2.95%, respectively (Figure 2(a)). At the genus level, the microbiota of both the NC and HP groups were dominated by *Lactobacillus* with relative abundances of 64.64% and 90.47%, respectively, while the microbiota of the treatment groups were dominated by *Bacteroides*, *Parabacteroides*, and *Barnesiella*, with average relative abundances of 25.97%, 11.49%, and 8.57%, respectively (Figure 2(b)).

#### 3.5.2. Alpha and Beta Diversities of the Gastric Microbiota

The diversity of gastric microbiota was evaluated through alpha diversity (Shannon and Simpson indexes) and beta diversity (PCoA and Anosim). As shown in Figures 2(c) and 2(d), the Shannon and Simpson indexes decreased in the HP group compared with the NC group (*P* = 0.026; *P* = 0.041), and no significant difference in alpha diversity indexes was observed between the HP group and the three treatment groups or among the three treated groups. The results of a PCoA based on unweighted UniFrac metrics are displayed in Figure 2(e); the correlated Anosim demonstrated a significant difference between the HP and NC groups (*R* = 0.235, *P* = 0.012), among the three treatment groups (RAC, RACS, and RACB) and HP group (*R* = 0.356, *P* = 0.009; *R* = 0.591, *P* = 0.003; and *R* = 0.376, *P* = 0.013), and between the RAC and RACS groups (*R* = 0.304, *P* = 0.011). No significant difference was found between the RACS and RACB groups (*R* = 0.135, *P* = 0.066). These findings showed that *H*. *pylori* infection significantly decreased the alpha diversity of gastric microbiota and changed gastric microbial composition. Moreover, the three treatments also had significant impact on gastric microbial structure, while alpha diversity of these three groups did not differ from the other groups.

#### 3.5.3. Composition Alteration in the Taxa of the Gastric Microbiota

**Figure 3:**
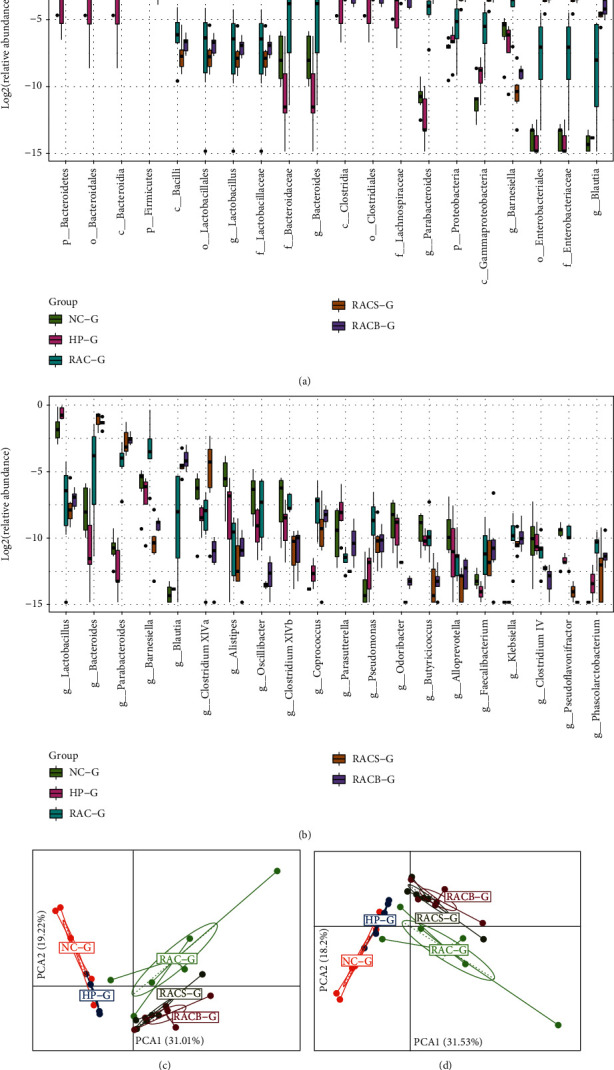
Composition alteration in the taxa of the gastric microbiota in mice. (a) Box plots with relative abundance of the top 20 different microbial taxa. (b) Box plots with relative abundance of the different microbial genera. (c) PCA based on the relative abundance of all differential taxa among five groups. (d) PCA based on the relative abundance of all differential genera among five groups. NC: natural control group; HP: *H*. *pylori* model group; RAC: standard triple therapy group; RACS: standard triple therapy+sucralfate group; RACB: bismuth-containing quadruple therapy group; G: gastric microbiota; p: phylum; c: class; o: order; f: family; g: genus.

To explore the distinct species among different groups, we used the Wilcoxon rank sum test and the Kruskal-Wallis rank sum test to conduct differential abundance analyses at all levels (phylum, family, class, order, and genus). The 102 differential abundant taxa of gastric microbiota were found among all groups, and 48 taxa were detected at the genus level. The top 20 different abundances of microbes among five groups are shown in Figure 3(a), and phylum level abundance was high for *Bacteroidetes* and *Proteobacteria*, while abundance for *Firmicutes* was lower in the three eradication groups. We found that the phylum *Bacteroidetes*, class *Bacteroidia*, order *Bacteroidetes*, family *Bacteroidaceae*, and genus *Bacteroides* were all more abundant in the therapy groups. As shown in Figure 3(b), the most affected specific genera in gastric microbiota were *Lactobacillus*, *Bacteroides*, *Parabacteroides*, *Barnesiella*, *Blautia*, *Clostridium XlVa*, and *Alistipes*. The PCA based on the relative abundance of all differential taxa or genera are shown in Figures 3(c) and 3(d), and we could not separate RACS from the RACB group, respectively, while the other groups were obviously distinct.

### 3.6. Alteration of Fecal Microbiota Composition after Eradication Therapy in *H*. *pylori*-Infected Mice

#### 3.6.1. Composition of Fecal Microbiota

**Figure 4:**
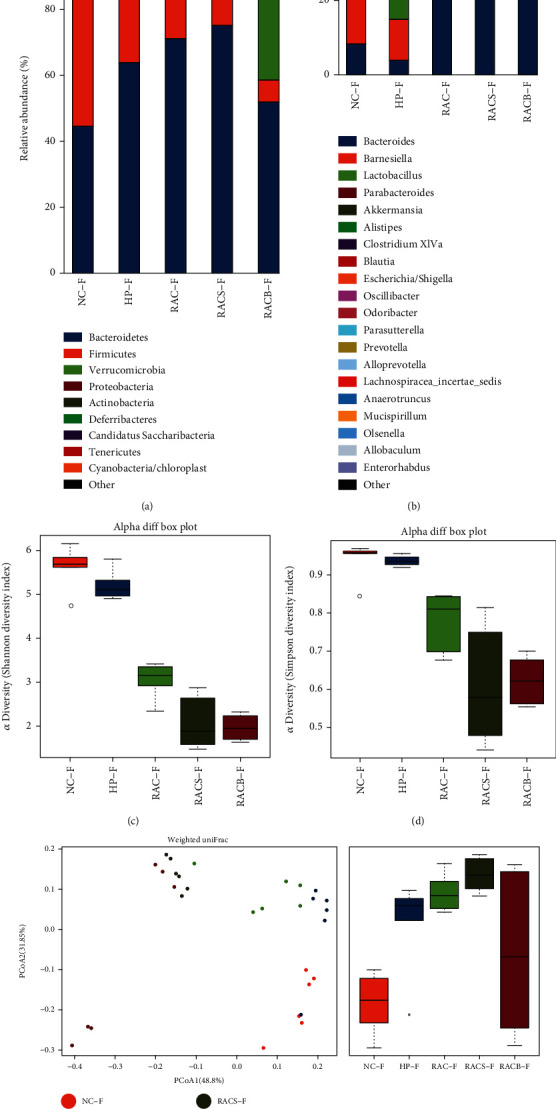
The composition, alpha diversity, and beta diversity of the fecal microbiome in mice. (a) Relative abundance distribution of major phyla of fecal microbiota in each group. (b) Relative abundance distribution of major genera of fecal microbiota in each group. (c) Shannon index of fecal microbiota based on the OTU counts. (d) Simpson index of fecal microbiota based on the OTU counts. (e) Weighted PCoA of fecal microbiota. NC: natural control group; HP: *H*. *pylori* model group; RAC: standard triple therapy group; RACS: standard triple therapy+sucralfate group; RACB: bismuth-containing quadruple therapy group; F: fecal microbiota; OTU: operational taxonomic unit; PCoA: principal coordinates analysis.

A total of 871,470 clean reads with an average of 29,049 reads per sample were generated from 30 fecal samples, and 445 OTUs at a 97% similarity level were generated afterwards. The most abundant phyla in feces were *Bacteroidetes*, *Firmicutes*, and *Proteobacteria* with average relative abundances of 61.36%, 26.47%, and 4.49%, respectively (Figure 4(a)). At the genus level, the fecal microbiota of both the NC and HP groups were dominated by *Alistipes*, *Lactobacillus*, and *Barnesiella*, while therapeutic groups were dominated by *Bacteroides*, *Parabacteroides*, *Akkermansia*, and *Barnesiella* (Figure 4(b)).

#### 3.6.2. Alpha and Beta Diversities of the Fecal Microbiota

The diversity of gut microbiota was evaluated as mentioned above. As shown in Figures 4(c) and 4(d), the Shannon and Simpson indexes showed no significant difference between the HP and NC groups (*P* = 0.180; *P* = 0.065). Those indexes decreased in the three treatment groups (RAC, RACS, and RACB) compared with the HP group. Furthermore, the Shannon and Simpson indexes of the RACS group were lower than the indexes of the RAC group (*P* = 0.009; *P* = 0.041), which were similar to the RACB group (*P* = 1.000; *P* = 0.699).

A PCoA based on weighted UniFrac metrics is shown in Figure 4(e), and the correlated Anosim demonstrated a significant difference between the HP and NC groups (*R* = 0.444, *P* = 0.016), among the three treatment groups (RAC, RACS, and RACB) and HP group (*R* = 0.587, *P*  = 0.003; *R* = 1.000, *P* = 0.002; and *R* = 0.820, *P* = 0.003), and between the RAC and RACS groups (*R* = 0.676, *P* = 0.008). No significant difference was found between the RACS and RACB groups (*R* = 0.272, *P* = 0.064). The data indicate that *H*. *pylori* infection changed fecal microbial composition while decreasing alpha diversity without significance. The three eradications obviously decreased alpha diversity of fecal microbiota and changed fecal microbial composition. Moreover, a similar transformation was found between the RACS and RACB groups.

#### 3.6.3. Composition Alteration in the Taxa of the Fecal Microbiota

**Figure 5:**
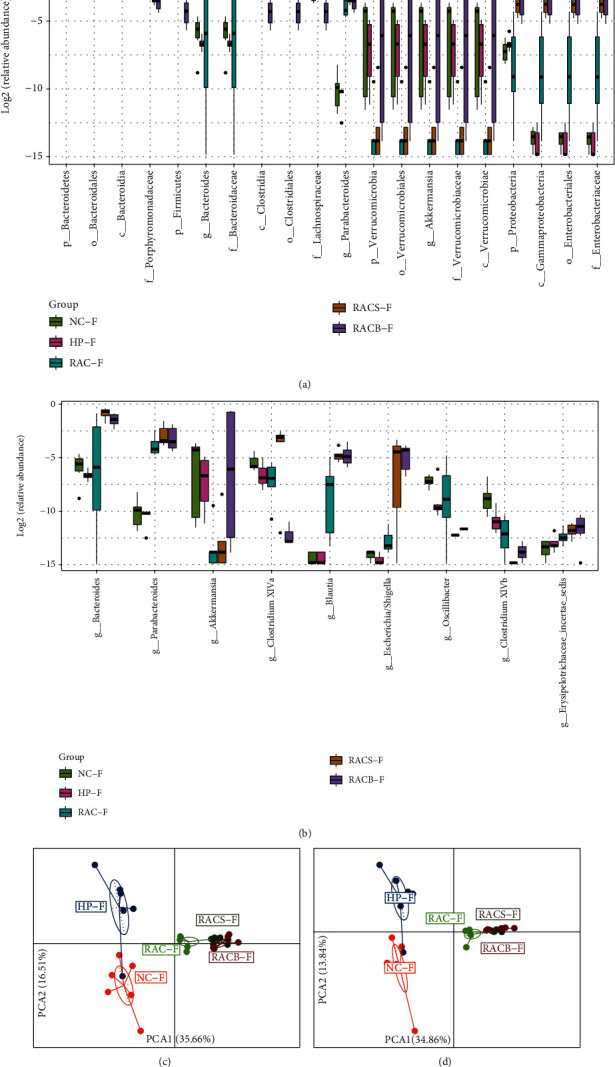
Composition alteration in the taxa of the fecal microbiota in mice. (a) Box plots with relative abundance of the top 20 different microbial taxa. (b) Box plots with relative abundance of the different microbial genera. (c) PCA based on the relative abundance of all differential taxa among five groups. (d) PCA based on the relative abundance of all differential genera among five groups. NC: natural control group; HP: *H*. *pylori* model group; RAC: standard triple therapy group; RACS: standard triple therapy+sucralfate group; RACB: bismuth-containing quadruple therapy group; F: fecal microbiota; p: phylum; c: class; o: order; f: family; g: genus.

We also evaluated differential abundance analyses at all levels as mentioned above. The 94 differential abundant taxa of fecal microbiota were found among all groups, and 40 taxa were found at the genus level. The top 20 different abundances of microbes among five groups in fecal microbiota are displayed in Figure 5(a), and phylum level abundance of *Bacteroidetes* was higher while *Firmicutes* was lower after *H*. *pylori* infection or eradication treatments. We found that all levels of *Bacteroidetes* were affected among all groups. The most affected specific genera in fecal microbiota were *Bacteroides*, *Parabacteroides*, *Akkermansia*, *Clostridium XlVa*, *Blautia*, *Escherichia*/*Shigella*, *Oscillibacter*, and *Clostridium XlVb* (Figure 5(b)). The PCA based on relative abundance of all differential taxa or genera are shown in Figures 5(c) and 5(d), and we also could not separate RACS from the RACB group, while the other groups were obviously distinct.

## 4. Discussion

In this study, we confirmed eradication efficacy of RACS in mice. RACS therapy also had a therapeutic effect in *H*. *pylori*-induced histological and ultrastructural damages, which was better than the RAC group and similar to the RACB group. The preliminary results indicated that the RACS regimen eradicated *H*. *pylori* infection effectively, which needs to be confirmed through further clinical studies.

Except for a direct bactericidal effect, the mechanisms of nonantibiotic drugs for eliminating *H*. *pylori* can be summarized as decreasing inflammatory factors, enhancing the mucosal barrier, transforming gastric microbiota, and so on^14^. After *H*. *pylori* infection, the local inflammation of gastric mucosa was caused by neutrophil granulocytes. A series of cytokines, such as IL-4, IL-6, IL-8, IL-10, and IL-12, were upregulated in gastric mucosal tissues [15]. These inflammatory factors formed a complicated network of immune inflammation to induce gastric mucosal damages [16]. The results of this study showed that IL-8 expression of the RACS group was significantly lower than that of the RAC group, while IL-10 and TNF-*α* of the RACS group was similar to those of the RAC group. These data showed that RACS therapy suppressed the inflammatory response by decreasing cytokines, especially by reducing IL-8 to ameliorate *H*. *pylori*-induced injury.

Tight junction proteins play an important role in the gastric epithelial barrier [17], including ZO-1 and Occludin. ZO-1 is a cytoskeletal protein of tight junction proteins [18], and Occludin is a transmembrane protein located on tight junction proteins [19]. Fan et al. [20] concluded that *H*. *pylori* infection dysregulated gastric epithelial barrier function by reducing ZO-1 and Occludin. Our results indicated that Occludin expression of the RACS group was elevated significantly compared with that of the RAC group, while no significant difference was noted in ZO-1 protein levels. These data suggested that RACS enhanced expression of the tight junction protein Occludin.

The main phyla of the gastric microbiota are *Proteobacteria*, *Firmicutes*, *Bacteroidetes*, and *Actinobacteria* in healthy individuals [21]. Additionally, the diversity of the human gastric microbiota decreased after *H*. *pylori* infection [22]. Our data revealed that the most abundant phyla of the mouse gastric microbiota were *Bacteroidetes*, *Firmicutes*, *Proteobacteria*, *Verrucomicrobia*, and *Actinobacteria*. *H*. *pylori* infection decreased alpha diversity and changed beta diversity, which was similar to the previous clinical study [22]. The treatment regimens markedly affected beta diversity while alpha diversity decreased insignificantly in mice. Li et al. concluded that alterations in gastric microbiota and reduction in bacterial diversity induced by *H*. *pylori* could be restored through antibiotic treatment in human beings [23]. However, our results showed that the gastric flora of the treatment groups was still significantly different compared to that of the normal mice, which indicated that the eradication drugs may affect the gastric flora, or the gastric flora needs a longer time to be restored after *H*. *pylori* eradication.

Antibiotic treatments can alter richness, diversity, and composition of gut microbiota in mice with a controlled environment [24]. This study showed that most abundant phyla of mouse fecal microbiota were *Bacteroidetes*, *Firmicutes*, and *Proteobacteria*, which were similar with human gut flora [25]. Additionally, the alpha diversity of gut microbiota in the HP group decreased compared with that in the NC group (*P* > 0.05) in mice, whereas an increase was found in a previous human research [25]. Three eradication therapies significantly altered diversity in mouse fecal microbiota. We observed disorders of *Bacteroidetes* and *Firmicutes* after *H*. *pylori* infection or eradication treatments in mice, which was a change associated with type 2 diabetes and Crohn's disease [26, 27]. Bacteroidetes has been reported to be associated with immunity and metabolism in primary biliary cirrhosis patients [28]. To our knowledge, the effects of different eradication regimens on gut microbiota composition have not been compared directly in patients or mice. We found the composition of mouse fecal microbiota after RACS was similar to the RACB group in PCA. It is noteworthy that genus *Akkermansia* of RACB mice was more prominent than that of RACS mice, although the difference was not significant, whereas *Akkermansia* decreased after bismuth-containing eradication in previous clinical studies [29, 30]. *Akkermansia* is a mucin-degrading beneficial bacterium, and it has been shown to reduce gut barrier disruption and insulin resistance [31, 32].

The limitation of this study is that we only discussed mouse gastrointestinal microbiota compositions without human results. Mice are used to easily control the diet or other environmental factors on microbial diversity of the intestinal tract and to relate this back to intervention measures. Although many common genera are shared in the human and murine intestines, these differ in abundance, which could weaken the application value of the mouse results [33]. Additionally, humans take different tablets before or after meals to eradicate *H*. *pylori*, while mice are given combined medicines simultaneously by oral gavage. Intragastric administration may make it easier for drugs to enter the gastrointestinal with a high dose and have obvious effects on microbial dysbiosis.

In conclusion, our results indicate that the RACS regimen might eradicate *H*. *pylori* effectively. RACS therapy has protective effects against *H*. *pylori*-induced histological and ultrastructural damages. The mechanisms of RACS for eliminating *H*. *pylori* included decreasing IL-8, enhancing Occludin, and transforming gastric microbiota. Moreover, RACS and RACB have similar effects on gastrointestinal flora.

## Figures and Tables

**Table 1:** RACS inhibited the overexpression of the IL-8 level induced by *H*. *pylori*.

Group	IL-8		IL-10		TNF-*α*	
	IHC	qPCR	IHC	qPCR	IHC	qPCR
NC	1.50 ± 0.22^a^	1.00 ± 0.12^a^	2.33 ± 0.33^a^	1.00 ± 0.14	2.33 ± 0.33^a^	1.00 ± 0.21^a^
HP	5.83 ± 0.31	2.14 ± 0.23	5.67 ± 0.49	1.42 ± 0.10	5.17 ± 0.40	1.76 ± 0.26
RAC	4.67 ± 0.21^ab^	1.94 ± 0.47^b^	4.33 ± 0.42	0.88 ± 0.25^a^	3.33 ± 0.21^a^	0.81 ± 0.17^a^
RACS	3.50 ± 0.22^a^	0.82 ± 0.18^a^	4.16 ± 0.31	0.39 ± 0.01^a^	3.17 ± 0.48^a^	0.66 ± 0.94^a^
RACB	3.83 ± 0.31^a^	0.99 ± 0.25^a^	5.17 ± 0.31	0.67 ± 0.25^a^	2.17 ± 0.31^a^	0.53 ± 0.55^a^

Mean ± SEM; compared with the HP group, ^a^*P* < 0.05; compared with the RACS group, ^b^*P* < 0.05.

**Table 2:** RACS enhanced tight junction protein Occludin expression.

Group	ZO-1	Occludin
NC	3.00 ± 0.37^a^	5.33 ± 0.42^a^
HP	1.17 ± 0.48	2.33 ± 0.21
RAC	2.5 ± 0.22	2.50 ± 0.22^b^
RACS	2.83 ± 0.31^a^	4.83 ± 0.48^a^
RACB	2.50 ± 0.34	5.00 ± 0.26^ab^

Mean ± SEM; compared with the HP group, ^a^*P* < 0.05; compared with the RACS group, ^b^*P* < 0.05.

## Data Availability

The data used to support the findings of this study are included within the article.

## Supplementary Materials

Supplementary Materials Supporting Information Table 1: primer sequences. Supporting Information Table 2: eradication effects of RACS on *H*. *pylori* infection, weight loss, and *H*. *pylori*-induced histological damage. Supporting Information Figure 1: RACS inhibited the *H*. *pylori*-induced overexpression of IL-8. Representative images of IL-8 (A), IL-10 (B), and TNF-*α* (C): (a) natural control group (NC), (b) *H*. *pylori* model group (HP), (c) standard triple therapy group (RAC), (d) standard triple therapy+sucralfate group (RACS), and (e) bismuth-containing quadruple therapy group (RACB). Scale bar: 200 *μ*m. Supporting Information Figure 2: RACS enhanced tight junction protein Occludin expression. Representative images of ZO-1 (A) and Occludin (B): (a) natural control group (NC), (b) *H*. *pylori* model group (HP), (c) standard triple therapy group (RAC), (d) standard triple therapy+sucralfate group (RACS), and (e) bismuth-containing quadruple therapy group (RACB). Scale bar: 200 *μ*m.

## References

[1] Malfertheiner P., Megraud F., O'Morain C. A. (2016). Management ofHelicobacter pyloriinfection—the Maastricht V/Florence Consensus Report. *Gut*.

[2] Xie C., Lu N. H. (2015). Review: clinical management of Helicobacter pylori infection in China. *Helicobacter*.

[3] Kentaro S. (2015). Screening of gastric cancer in Asia. *Best Practice & Research. Clinical Gastroenterology*.

[4] Thung I., Aramin H., Vavinskaya V. (2016). Review article: the global emergence of Helicobacter pylori antibiotic resistance. *Alimentary Pharmacology & Therapeutics*.

[5] Gao W., Cheng H., Hu F. (2010). The evolution of helicobacter pylori antibiotics resistance over 10 years in Beijing, China. *Helicobacter*.

[6] Liu W. Z., Xie Y., Lu H. (2018). Fifth Chinese national consensus report on the management of Helicobacter pylori infection. *Helicobacter*.

[7] Wang Y., Wang B., Lv Z. F. (2014). Efficacy and safety of ecabet sodium as an adjuvant therapy for Helicobacter pylori eradication: a systematic review and meta-analysis. *Helicobacter*.

[8] Cui M. H., Wei H., Lei X. Y., Dai L. N., Ma Z. L. (2014). Efficacy of compound allantoin containing quadruple regimen in the treatment of chronic gastritis with Helicobacter pylori infection. *Chin J Dig*.

[9] Wang T. T., Zhang Y. M., Zhang X. Z. (2013). Jinghuaweikang gelatin pearls plus proton pump inhibitor-based triple regimen in the treatment of chronic atrophic gastritis with Helicobacter pylori infection: a multicenter, randomized, controlled clinical study. *Zhonghua Yi Xue Za Zhi*.

[10] Li Q., Wang N. N., Hu F. L., Li C., Li J., Yang G. B. (2016). Study of compound bismuth and magnesium granules on clearance of helicobacter pylori infection in KM mice. *International Journal of Clinical and Experimental Medicine*.

[11] Tan B., Luo H. Q., Xu H. (2017). Polaprezinc combined with clarithromycin-based triple therapy for Helicobacter pylori-associated gastritis: a prospective, multicenter, randomized clinical trial. *PLoS One*.

[12] Fang S., Sheng J. Q., Jin P., Li S. J. (2017). Effect of standard triple and quadruple classic therapy combined with hydrotalcite in Helicobacter pylori eradication of troops. *Chin J Gastroenter Hepatol*.

[13] Chai X. (2013). The clinical efficacy of sucralfate suspensiod gel. *Scientific & Technical Information of Gansu*.

[14] Hu F. L. (2012). A new approach to the treatment of Helicobacter pylori infection. *Natl Med J China*.

[15] Hosseini M. E., Oghalaie A., Habibi G. (2010). Molecular detection of host cytokine expression in helicobacter pylori infected patients via semi-quantitative RT-PCR. *Indian Journal of Medical Microbiology*.

[16] Walduck A., Andersen L. P., Raghavan S. (2015). Inflammation, immunity, and vaccines for Helicobacter pylori infection. *Helicobacter*.

[17] Wroblewski L. E., Shen L., Ogden S. (2009). Helicobacter pylori dysregulation of gastric epithelial tight junctions by urease-mediated myosin II activation. *Gastroenterology*.

[18] Müller S. L., Portwich M., Schmidt A. (2005). The tight junction protein Occludin and the adherens junction protein alpha-catenin share a common interaction mechanism with ZO-1. *The Journal of Biological Chemistry*.

[19] Osanai M., Murata M., Nishikiori N., Chiba H., Kojima T., Sawada N. (2007). Occludin-mediated premature senescence is a fail-safe mechanism against tumorigenesis in breast carcinoma cells. *Cancer Science*.

[20] Fan Y., Wang Z., Guan Y., Han W. W., Jiang Z. D., Wang J. S. (2017). Expressions of tight junction protein Occludin and ZO-1 in patients with chronic gastritis of Helicobacter pylori infection. *Chinese Journal of Gastroenterology and Hepatology*.

[21] Bik E. M., Eckburg P. B., Gill S. R. (2006). Molecular analysis of the bacterial microbiota in the human stomach. *Proc Natl Acad Sci USA*.

[22] Andersson A. F., Lindberg M., Jakobsson H., Bäckhed F., Nyrén P., Engstrand L. (2008). Comparative analysis of human gut microbiota by barcoded pyrosequencing. *PLoS One*.

[23] Li T. H., Qin Y., Sham P. C., Lau K. S., Chu K. M., Leung W. K. (2017). Alterations in Gastric Microbiota After *H. Pylori* Eradication and in Different Histological Stages of Gastric Carcinogenesis. *Scientific Reports*.

[24] Antonopoulos D. A., Huse S. M., Morrison H. G., Schmidt T. M., Sogin M. L., Young V. B. (2009). Reproducible community dynamics of the gastrointestinal microbiota following antibiotic perturbation. *Infection and Immunity*.

[25] Gao J. J., Zhang Y., Gerhard M. (2018). Association between gut microbiota and Helicobacter pylori-related gastric lesions in a high-risk population of gastric cancer. *Frontiers in Cellular and Infection Microbiology*.

[26] Larsen N., Vogensen F. K., van den Berg F. W. J. (2010). Gut microbiota in human adults with type 2 diabetes differs from non-diabetic adults. *PLoS One*.

[27] Man S. M., Kaakoush N. O., Mitchell H. M. (2011). The role of bacteria and pattern-recognition receptors in Crohn's disease. *Nature Reviews. Gastroenterology & Hepatology*.

[28] Lv L. X., Fang D. Q., Shi D. (2016). Alterations and correlations of the gut microbiome, metabolism and immunity in patients with primary biliary cirrhosis. *Environmental Microbiology*.

[29] Hsu P. I., Pan C. Y., Kao J. Y. (2018). Helicobacter pylori eradication with bismuth quadruple therapy leads to dysbiosis of gut microbiota with an increased relative abundance of Proteobacteria and decreased relative abundances of Bacteroidetes and Actinobacteria. *Helicobacter*.

[30] Yildiz S. S., Yalinay M., Karakan T. (2018). Bismuth-based quadruple Helicobacter pylori eradication regimen alters the composition of gut microbiota. *Le Infezioni in Medicina*.

[31] Everard A., Belzer C., Geurts L. (2013). Cross-talk between Akkermansia muciniphila and intestinal epithelium controls diet-induced obesity. *Proceedings of the National Academy of Sciences of the United States of America*.

[32] Chelakkot C., Choi Y., Kim D.-K. (2018). Akkermansia muciniphila-derived extracellular vesicles influence gut permeability through the regulation of tight junctions. *Experimental & Molecular Medicine*.

[33] Hugenholtz F., de Vos W. M. (2018). Mouse models for human intestinal microbiota research: a critical evaluation. *Cellular and Molecular Life Sciences*.

