# Supplementary material for: Efficacy of Sucralfate-Combined Quadruple Therapy on Gastric Mucosal Injury Induced by Helicobacter pylori and Its Effect on Gastrointestinal Flora
Source: Biomed Res Int. 2020 Aug 31;2020:4936318. doi: 10.1155/2020/4936318 (PMC7479470; doi:10.1155/2020/4936318)
Supplement: Supplementary Materials — Supporting Information Table 1: primer sequences. Supporting Information Table 2: eradication effects of RACS on H. pylori infection, weight loss, and H. pylori-induced histological damage. Supporting Information Figure 1: RACS inhibited the H. pylori-induced overexpression of IL-8. Representative images of IL-8 (A), IL-10 (B), and TNF-α (C): (a) natural control group (NC), (b) H. pylori model group (HP), (c) standard triple therapy group (RAC), (d) standard triple therapy+sucralfate group (RACS), and (e) bismuth-containing quadruple therapy group (RACB). Scale bar: 200 μm. Supporting Information Figure 2: RACS enhanced tight junction protein Occludin expression. Representative images of ZO-1 (A) and Occludin (B): (a) natural control group (NC), (b) H. pylori model group (HP), (c) standard triple therapy group (RAC), (d) standard triple therapy+sucralfate group (RACS), and (e) bismuth-containing quadruple therapy group (RACB). Scale bar: 200 μm. [file 4936318.f1.docx]

Supporting Information Table 1. Primer sequences.

| Primer names | Primer sequences (5'to3') |
| --- | --- |
| *H. pylori*- Forward Primer | TGGCGTGTCTATTGACAGCGAAC |
| *H. pylori*- Reverse Primer | CCTGCTGGGCATACTTCACCATG |
| IL8- Forward Primer | TCGAGACCATTTACTGCAACAG |
| IL8- Reverse Primer | CATTGCCGGTGGAAATTCCTT |
| IL-10- Forward Primer | GCTCTTACTGACTGGCATGAG |
| IL-10- Reverse Primer | CGCAGCTCTAGGAGCATGTG |
| TNFα- Forward Primer | CAGGCGGTGCCTATGTCTC |
| TNFα- Reverse Primer | CGATCACCCCGAAGTTCAGTAG |
| GAPDH- Forward Primer | AGGTCGGTGTGAACGGATTTG |
| GAPDH- Reverse Primer | TGTAGACCATGTAGTTGAGGTCA |

Supporting Information Table 2. Eradication effects of RACS on *H.pylori* infection, weight loss, *H.pylori*-induced histological damage.

| Group | Eradication rates of *H. pylori* by IHC | Eradication rates of *H. pylori* by PCR | HE scores |
| --- | --- | --- | --- |
| NC | 0/6(0.00%) | 0/6(0.00%) | 1.17±0.17 ^a^ |
| HP | 1/12(8.33%) | 3/12(25.00%) | 3.42±0.19 |
| RAC | 8/12(66.67%) | 10/12(83.33%) | 2.67±0.31 ^a^ |
| RACS | 10/12(83.33%) | 11/12(91.67%) | 1.67±0.14 ^a^ |
| RACB | 11/12(91.67%) | 11/12(91.67%) | 2.09±0.25 ^ab^ |

mean±SEM; n(%); Compared with HP group，^a^*P*<0.05；Compared with RACS group，^b^*P*<0.05.


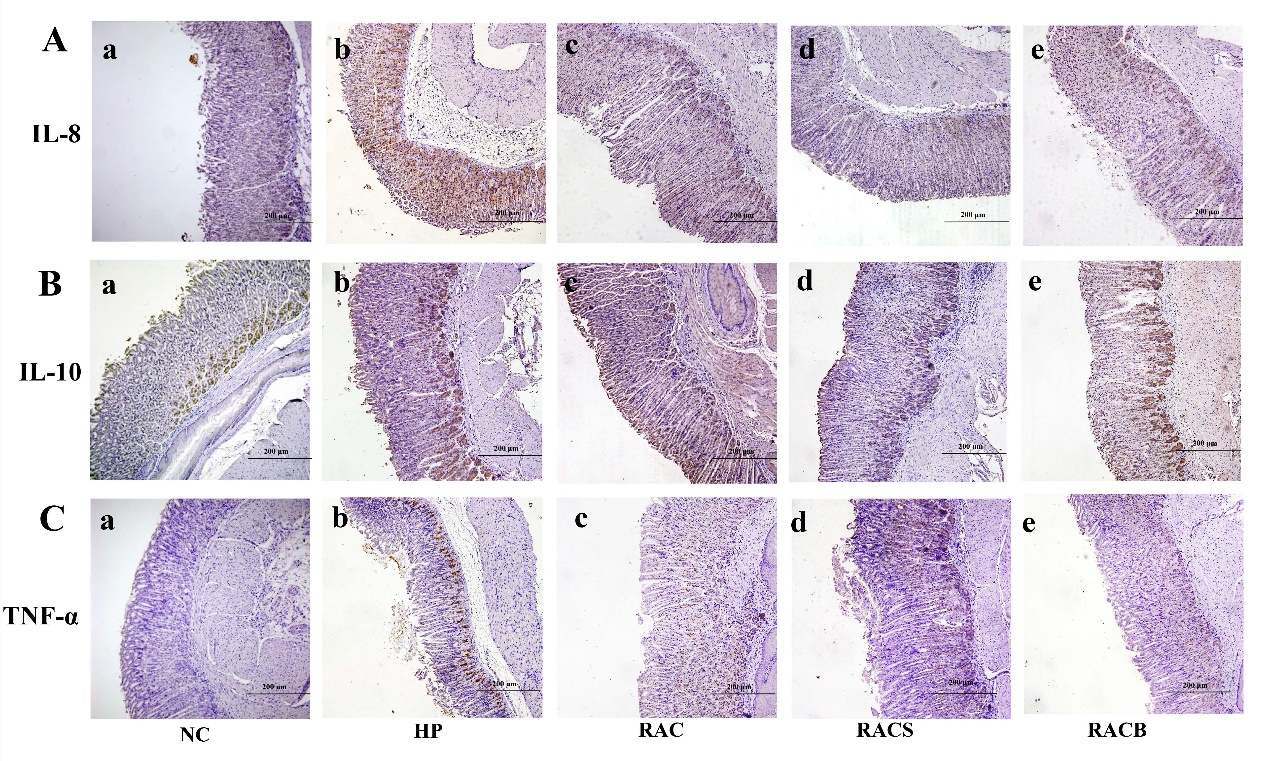


Supporting Information Figure 1. RACS inhibited the *H.pylori*-induced overexpression of IL-8. Representative images of IL-8(A), IL-10(B) and TNF-α(C) were shown: (a) natural control group (NC), (b) *H.pylori* model group (HP), (c)standard triple therapy group (RAC), (d) standard triple therapy+sucralfate group (RACS), (e) bismuthcontained quadruple therapy group (RACB). Scale bar: 200 μm.


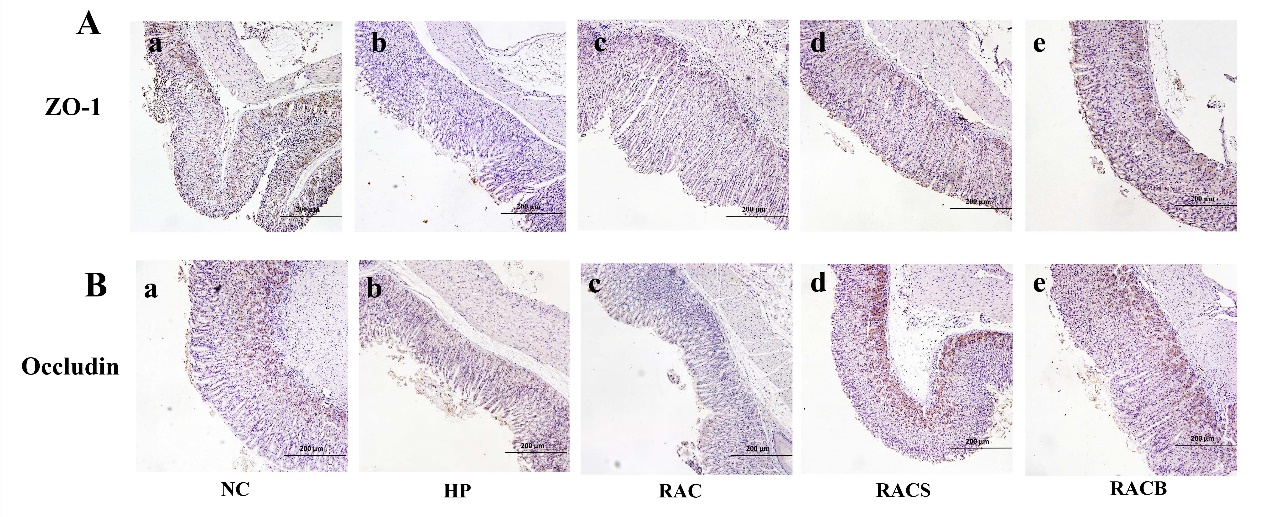


Supporting Information Figure 2. RACS enhanced tight junction protein Occludin expression. Representative images of ZO-1(A) and Occludin(B) are: (a) natural control group (NC), (b) *H.pylori* model group (HP), (c) standard triple therapy group (RAC), (d) standard triple therapy+sucralfate group (RACS), (e) bismuth-contained quadruple therapy group (RACB). Scale bar: 200 μm.
